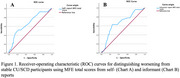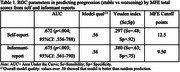# Discrimination between stability and worsening in participants without objective cognitive impairment using Memory Failure of Everyday (MFE) questionnaire total scores: Comparison of Self‐ and Informant‐reports

**DOI:** 10.1002/alz70857_105061

**Published:** 2025-12-25

**Authors:** Lucía Pérez‐Blanco, Fátima Fernández‐Feijoo, Sonali Arora, Xana Cid‐Mejías, Ana Nieto‐Vieites, Alba Felpete, Sonia Valladares‐Rodríguez, Arturo X. Pereiro Rozas

**Affiliations:** ^1^ Applied Cognitive Neuroscience and Psychogerontology group, Health Research Institute of Santiago de Compostela (IDIS), Santiago de Compostela, Spain; ^2^ Departamento de Psicoloxía Evolutiva e da Educación, Universidade de Santiago de Compostela, Santiago de Compostela, Galicia, Spain; ^3^ Instituto de Psicoloxía (IPsiUS), Universidade de Santiago de Compostela, Santiago de Compostela, Galicia, Spain; ^4^ Departamento de Electrónica e Computación, Escola Politécnica Superior de Inxeniería, Universidade de Santiago de Compostela, Santiago de Compostela, Spain

## Abstract

**Background:**

Cognitive complaints, particularly those focused on memory, has been proposed as a factor risk for progression to objective cognitive impairment and dementia (Nosheny et al., 2022).

**Method:**

The Memory Failure of Everyday (MFE), a 28‐item questionnaire to assess memory complaints (Sunderland et al.1984), was administered to cognitively unimpaired participants using a 3‐response scale (i.e., 0=never/rare; 1=little/occasionally; 2=very often). Total scores for MFE were obtained at baseline for both participants and informants.

One‐hundred and thirty in older adults (Age: *M* = 63.11; *SD* = 7.94; 75.38% women) without objective cognitive impairment (i.e., CU, SCD) and their correspondent informants (*N* = 127; 50% women) from the CompAS were included in the study.

Participants were evaluated longitudinally on three occasions (range = 47‐77 months; *M* = 59.86; *SD* = 7.36) and progressors to MCI or dementia according to the criteria of Albert et al. (2011) were identified.

Two ROC analyses were performed to explore the value of participant and informant MFE total scores to discriminate between worsening and stable participants using, respectively, self‐ and informant‐reports.

**Results:**

ROC models for self‐ and informant‐report achieved significance. Area Under the Curve was similar and modest for MFE total scores from both self‐ and informant‐report (see Figure 1), although the specificity (self: .92; informant: .75) and the sensitivity (self: .48; informant: .63) values were somewhat different (see Table 1).

**Conclusion:**

MFE total scores from self‐ and informant‐report showed similar AUC values. Self‐ and Informant‐report were more accurate in identifying stable participants (specificity). However, informant report showed greater accuracy in identifying worsening participants (sensitivity) and self‐report showed greater accuracy in identifying stable participants (specificity).